# Molecular Surface Remeshing with Local Region Refinement

**DOI:** 10.3390/ijms19051383

**Published:** 2018-05-06

**Authors:** Dawar Khan, Dong-Ming Yan, Sheng Gui, Benzhuo Lu, Xiaopeng Zhang

**Affiliations:** 1 National Laboratory of Pattern Recognition, Institute of Automation, Chinese Academy of Sciences, Beijing 100190, China; dawar@ia.ac.cn (D.K.); xiaopeng.zhang@ia.ac.cn (X.Z.); 2 University of Chinese Academy of Sciences, Beijing 100049, China; shenggui@lsec.cc.ac.cn (S.G.); bzlu@lsec.cc.ac.cn (B.L.); 3 National Center for Mathematics and Interdisciplinary Sciences, State Key Laboratory of Scientific and Engineering Computing, Academy of Mathematics and Systems Science, Chinese Academy of Sciences, Beijing 100190, China

**Keywords:** molecular surface mesh, molecular simulation, meshing quality, finite element method

## Abstract

Molecular surface mesh generation is a prerequisite for using the boundary element method (BEM) and finite element method (FEM) in implicit-solvent modeling. Molecular surface meshes typically have small angles, redundant vertices, and low-quality elements. In the implicit-solvent modeling of biomolecular systems it is usually required to improve the mesh quality and eliminate low-quality elements. Existing methods often fail to efficiently remove low-quality elements, especially in complex molecular meshes. In this paper, we propose a mesh refinement method that smooths the meshes, eliminates invalid regions in a cut-and-fill strategy, and improves the minimal angle. We compared our method with four different state-of-the-art methods and found that our method showed a significant improvement over state-of-the-art methods in minimal angle, aspect ratio, and other meshing quality measurements. In addition, our method showed satisfactory results in terms of the ratio of regular vertices and the preservation of area and volume.

## 1. Introduction

Surface meshes are typically used in modeling, animation, numerical simulation, and many other applications. Molecular surface meshes play a vital role in the study of evolution and interaction of molecules and in measuring their areas and volumes [1,2]. These meshes are used in various fields of computational biology, such as protein folding, structure prediction, docking and implicit-solvent modeling. However, these meshes are generated in raw form, thereby containing low-quality elements. Such raw meshes with low-quality elements are difficult to be directly used in downstream applications. Recent development in mathematical modeling and simulation of biomolecules, especially in implicit solvent modeling, demands a proper refinement of these molecular meshes to remove low-quality elements and improve meshing quality [3].

The molecular Gaussian surface is represented by the blending of a set of Gaussian functions. Various algorithms have been developed to triangulate and render molecular surfaces. TMSmesh [4,5] is a manifold triangular meshing framework used for meshing large Gaussian molecular surfaces. TMSmesh can handle a number of tasks, such as overlapping, gap filling, and seed selection, that need to be considered in traditional continuation methods. It succeeds in surface mesh generation for biomolecules containing more than one million atoms. TMSmesh generates meshes with satisfactory quality in terms of uniformity and manifoldness.The output mesh preserves the geometry and features (topology, area and volume, and local curvature) of the input mesh. However, the computational efficiency still needs to be improved. In this regard, Liu et al. [6] proposed an improved version i.e., TMSmesh 2.0. Their results show that TMSmesh 2.0 is robust, efficient, and more than thirty times faster as compared to the previous version [4].

Quality requirements in mesh processing which defines the class of acceptable and supported models varies from application to application [7]. Molecular remeshing has its own challenges and issues, such as raw input meshes, very small angles, and complex geometry. Different methods have been proposed to handle various issues in molecular surface remeshing. ISO2mesh [8] is a mesh processing toolbox used for mesh smoothing and tetrahedral mesh generation. It can be used for molecular mesh smoothing, but defects such as self-intersecting triangles and small angle triangles remain in its results. Recently, Liu et al. [9] proposed a method called SMOPT to improve molecular remeshing with the removal of redundant vertices and self-intersecting triangles. However, there is no considerable improvement in minimal and maximal angles in SMOPT results. The literature study shows that the existing methods for molecular refreshing somehow fail to handle maximal and minimal angles, self-intersecting triangles, redundant vertices, and other defects. Therefore, efficient methods for quality improvement of molecular meshes are demanded.

In this paper, we proposed a simple method to improve molecular surface meshes. The proposed method starts with real-time adaptive remeshing (RAR) [10] initialization followed by aspect ratio improvement. Furthermore, a cut-and-fill method is applied to remove invalid regions and refill them. The newly filled regions are further improved via edge split, edge collapse, and vertex translation with the condition of minimal angle improvement. We compared our results with two recent methods including SMOPT and ISO2mesh and found a significant improvement in the meshing quality. The results reveal that our method preserves the volume and area of the input mesh, and has a solvation energy similar to SMOPT, removes redundant vertices, and eliminates small angles (i.e., <30${}^{\circ }$). The main contributions of this study are as follows:

- We propose a mesh refinement method for smoothing molecular surface meshes and improve the minimal and maximal angles to an angle bound [30${}^{\circ }$, 120${}^{\circ }$].
- A cut-and-fill strategy is used to carefully remove the invalid regions with redundant vertices and/or small angles.
- A global smoothing method is used to improve aspect ratio, and local operators are used to refine the newly filled holes and improve the minimal angle.

## 2. Related Work

Molecular surfaces have various definitions based on their molecular structure. In a recent study [11], Chen and Lu summarized molecular surfaces, including van der Waals (VDW) surfaces, solvent accessible surfaces (SASs), solvent excluded surfaces (SESs), molecular skin surfaces, minimal molecular surfaces, and Gaussian surfaces. In this section, we briefly review the existing methods in molecular surface meshing. We recommend books [12] and review articles [11,13,14] for detailed studies. Alliez et al. [13] reviewed surface remeshing techniques generally used in computer graphics and geometry processing applications. Chen and Lu [11] conducted a review specific in molecular surface remeshing. Similarly, Bade et al. [14] compared state-of-the-art methods of medical mesh smoothing.

In computer graphics, numerous surface remeshing methods have been proposed. These methods can be classified as mesh-simplification-based methods [15,16], Delaunay insertion methods [17], advancing-front-based methods [18], field-based approaches [19,20], and local-operator-based mesh optimization [10,21]. In addition, global optimization methods which include parametrization-based methods [22,23], discrete clustering [24], and direct 3D optimization [25,26,27,28] are also available. Furthermore, segmentation-based meshing are also used, where input meshes are segmented prior to remeshing, which helps to preserve sharp features [29,30]. In terms of implicit feature preservation, several approaches exhibit efficient feature functions [24,31]. Laplacian smoothing [32] is the simplest method, which involves moving each vertex to the central position of its neighbor. Equation (1) computes the new position $v_{f}$ for a free vertex $v_{i}$ as the median of the positions of the *n* vertices $q_{1},q_{2},q_{3},\ldots ,q_{n}$ in its one-ring neighborhood.

(1) $$v_{f}=\frac{1}{n}\sum_{j=1}^{n}q_{j}.$$

Taubin [33] proposed a LowPass filter, which combines two Laplace like filters, one with positive weight and one with negative weight. The Taubin method [33] computes new position $p_{f}$ from old position $p_{i}$ using Equation (2):

(2) $$p_{f}=p_{i}+\lambda \sum_{j=1}^{n}\omega \left(q_{j}-p_{i}\right).$$

Here the weighting factor ω is commonly used as $\omega =\frac{1}{n}$. λ is the weighting factor, which is replaced by another weighting factor μ=−(λ+ϵ) with a small value ϵ=0.02. ϵ is used to set the value of μ a bit smaller than −λ. These two weighting factors including λ and μ are alternatively applied for backward translation [33].

Recent methods show a significant improvement in minimal and maximal angles and meshing quality for graphical models. For example, centroidal Voronoi tessellation (CVT) [34,35] smooths meshes by translating vertices to new positions which optimize an energy function. Real-time adaptive remeshing (RAR) [10] is a high-quality adaptive remeshing approach that is suitable for real-time applications. Mansouri and Ebrahimnezhad proposed a curvature-adapted subdivision method [36], which minimizes the distortion error and improves the aspect ratio (AR). Yan and Wonka [37] proposed a non-obtuse remeshing method using additional operators with CVT to avoid short Voronoi edges. In this manner, they remove small angles (<30${}^{\circ }$) and obtuse angles. However, for noisy meshes, their method fails to achieve the desired angle bound (i.e., [30${}^{\circ }$, 90${}^{\circ }$]). Recently, Hu et al. [31] proposed a remeshing method with common local edge-based operators (edge split, edge collapse, and edge flip and edge split) and vertex smoothing. They generated results with a minimal angle higher than 35${}^{\circ }$ with feature preservation. These approaches can efficiently handle common graphical models, such as CAD models and man-made objects. However, for molecular remesing, these methods are not directly applicable. Tiny triangles (with zero or near-zero degree angles), redundant vertices, feature preservation, and complex geometry are the specific challenges with molecular surface meshes where the existing remeshing methods often fail.

The removal of defects from a raw mesh is also an interesting topic in mesh processing. Ju presented PolyMender [38], a simple yet robust method for repairing polygonal models. The algorithm generates closed surface meshes, repairing all existing defects in the input model with features preservation. MeshFix [39] is another tool used to convert raw digitized polygons to clean mesh avoiding holes, non-manifold elements, and degenerate or intersecting elements. Experiments show that MeshFix provides results that have a higher visual quality, are more accurate, and add fewer new triangles compared to their previous methods. Attene et al. [7] summarized typical defects that make a 3D model unsuitable for different applications and reviewed the existing refreshing techniques.

Meshlab [40] is another tool used for surface repair, reconstruction, and smoothing purposes. It enables the implementation of several state-of-the-art methods such as mesh smoothing methods [33,41,42,43] and several modules for mesh cleaning and repairing. Similarly, Graphite [44] is also used for surface smoothing, remeshing, 3D modeling, and surface repairing purposes. It is used to visualize holes and non-manifold configurations, to fill holes, and to remove self-intersections.

In the molecular modeling community, Decherchi and Rocchia [45] proposed a ray-casting method for the triangulation of complex manifold surfaces in the nano-bioscience field. They summarized various applications of molecular surfaces in implicit solvent modeling and simulations using the boundary element method (BEM) and the finite element method (FEM). TMSmesh [4,5] generates molecular surface meshes for molecules. TMSmesh 2.0 can efficiently generate manifold surface meshes for biomolecules with more than one million atoms with shape and feature preservations [46]. The Molecular Finite Element Solver (mFES) [47] is a tool that uses tetrahedral finite elements to calculate electrostatic potentials of large molecular systems.

ISO2mesh [8] is a free Matlab/octave-based toolbox used for mesh generation and processing. It is used to create tetrahedral meshes from surface meshes and 3D binary and gray-scale volumetric images such as segmented MRI/CT scans. ISO2mesh is also used for molecular mesh smoothing, but it fails to handle self-intersecting triangle pairs and small angle triangles. Liu et al. [9] proposed an algorithm called SMOPT for molecular surface remeshing. They used local modifications on the mesh to improve the mesh quality, eliminate redundant vertices, avoid non-manifold errors, and remove intersecting triangles. For mesh smoothing, SMOPT has improved Laplacian smoothing, which is given in Equation (3).

(3) $$P_{i}=\left(1-\beta \right)q_{i}+\frac{\beta }{N_{i}}\sum_{j=1}^{N_{i}}q_{j}$$

where β∈[0,1] is the parameter to control the rate of smoothing, $N_{i}$ represents the number of vertices in the one ring, and $q_{j}$ represent the *j*th adjacent vertex in the one ring of the *i*th vertex. SMOPT results show a significant improvement in the mesh quality. Still, there are very small angles which destroy the quality of the triangles.

## 3. Preliminaries & Definitions

### 3.1. Gaussian Surface

A level set of the summation of Gaussian kernel functions (Equation (4)) defines the Gaussian surface.

(4) $$\left\{\vec{x}\in R^{3},\phi \left(\vec{x}\right)=c\right\}$$

where $\phi (\vec{x})$ is defined in Equation (5), and *c* is the isovalue which controls the volume.

(5) $$\phi \left(\vec{x}\right)=\sum_{i=1}^{N}e^{-d(∥\vec{x}-\vec{x_{i}}{∥}^{2}-r_{i}^{2})}$$

where $\vec{x_{i}}$ is the location, $r_{i}$ is the radius of the *i*th atom, *N* represents the number of atoms in the molecule, and *d* represent the decay rate of the Gaussian kernel. As decay rate decreases, molecules show more geometric details [46].

### 3.2. Molecular Surface Mesh Generation

A benchmark of molecular structures in PDB (protein data bank) and PQR formats can be found in the website http://doi.org/10.6084/m9.figshare.6143834, which was used in the previous tests. In the benchmark, some structures are directly taken from PDB IDs, and some are mixed or modified (used in molecular dynamics simulations). Based on the PDB structure, the corresponding PQR files are generated using pdb2pqr software [48]. In PQR format, the occupancy and temperature factor columns of a PDB file are replaced with charge *Q* and radius *R*, respectively. These files are compatible with several popular computational biology tools [48]. PQR files are given to the TMSmesh [4,6] for surface mesh generation. The surface mesh generated by TMSmesh 2.0 typically has a number of zero degree angles and redundant vertices, which needs further refinement. For example, SMOPT, ISO2mesh, and our current method are used for mesh improvement at this stage. After the surface mesh is improved, the next step is tetrahedralization for volume mesh generation via TetGen [49].

### 3.3. Application to a Boundary Element Simulation of Poisson–Boltzmann Electrostatics

Electrostatics is considered an important factor in understanding the interactions and dynamics of molecular systems in solutions. One commonly used continuum model for describing the electrostatic effects of the solvent outside molecules is the Poisson–Boltzmann (PB) equation [50], which is given in Equation (6):

(6) $$-\nabla \cdot \left(\epsilon \nabla \phi \right)+\lambda k^{2}\sinh\left(\phi \right)=\rho^{f}\left(r\right)$$

where λ is 0 in $\Omega_{m}$ and 1 in $\Omega_{s}$. In the case of small electrostatic potentials, Equation (7) is used, which is called the linearized Poisson–Boltzmann (LPB):

(7) $$-\nabla \cdot \left(\epsilon \nabla \phi \right)+\lambda k^{2}\phi =\rho^{f}\left(r\right).$$

**Figure 1:**
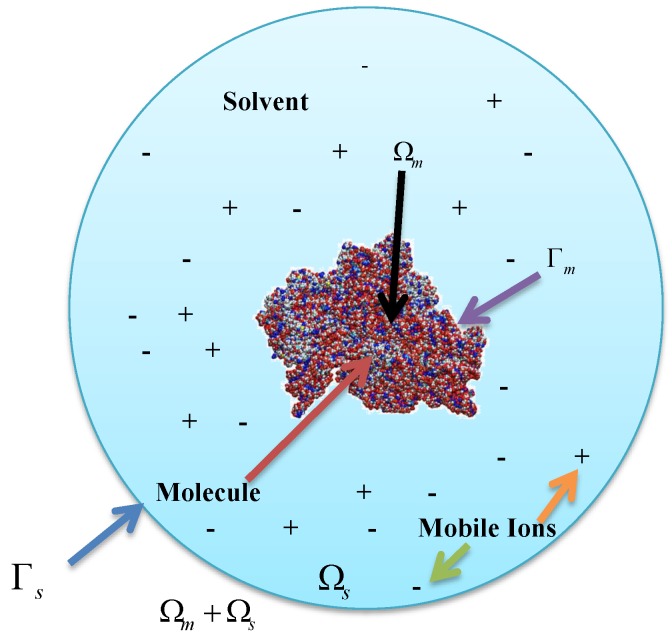
Schematic illustration of the computational domain.

Figure 1 illustrates a biomolecular solution system occupying domain Ω with a smooth boundary ∂Ω. Domain $\Omega_{s}$ denotes the solvent region that contains several diffusing species and domain $\Omega_{m}$ denotes the biomolecular region. Here, $\Omega =\Omega_{s}⋃\Omega_{m}$ and denotes the boundary of $\Omega_{m}$. ϕ(r) is the electrostatic potential, $\rho^{f}\left(r\right)=\sum_{i=1}^{s}q_{i}\delta \left(r-r_{i}\right)$ is an ensemble of the atomic point charges $q_{i}$ located at $r_{i}$ inside $\Omega_{m}$ (i=1,2,⋯,s), s is the number of atoms, δ(·) is the Dirac Delta function, and ϵ(r) is the dielectric coefficient distribution function.

In most practically used PB models in computational chemistry and biophysical communities, the dielectric coefficient is usually taken as piecewise constants dependent on regions as follows:

$$\epsilon =\left\{\begin{array}{cc} \epsilon_{m}, & in\;\Omega_{m}\\ \epsilon_{s}, & in\;\Omega_{s} \end{array}\right..$$

Molecular surface/volume mesh is required for boundary element/finite element types of simulations. The boundary element method is an accurate numerical method to solve the (linearized) PBE. Along this research direction, Lu et al. [50] have developed a highly efficient algorithm and software called AFMPB. Qualified molecular surface mesh generation is a demanding and very challenging task, especially for large molecules. Our previously developed method, TMSmesh is able to efficiently generate a manifold surface triangular mesh for arbitrarily large molecules. However, the mesh quality from TMSmesh is sometimes not sufficient for further volume mesh generation and/or for convergence of numerical simulations using BEM/FEM. This is why we will present a remeshing method in Section 4 to further improve surface mesh quality.

### 3.4. Surface Features Preservation

Similar to other surface generation software, such as the most commonly used MSMS [51], the surface mesh generated by TMSmesh2.0 preserves molecular surface features and thus can be applied to the molecular visualization and analysis of surface area, topology, and volume in computational structure biology and structural informatics. This new method also comes from TMSmesh2.0, so its details are similar to those of our method.

## 4. The Proposed Molecular Remeshing Method

Our method starts with RAR remeshing followed by aspect ratio improvement. After this, local operators cut, fill, and smooth are repeated until a high-quality mesh is generated. Algorithm 1 represents the main modules of our method, which is described below. Here $M_{i}$ represents input mesh, $M_{R}$ represents the intermediate mesh, and $M_{f}$ is the output mesh.

**Algorithm 1** Molecular Remeshing ($M_{i}$)
1:
$M_{R}\leftarrow RAR\left(M_{i}\right)$
2:$M_{R}\leftarrow ImproveAspectRatio\left(M_{R}\right)$ // Algorithm 23:
$M_{R}\leftarrow CutInvalidRegions\left(M_{R},15^{\circ }\right)$
4:
$M_{R}\leftarrow FillHoles\left(M_{R}\right)$
5:$M_{R}\leftarrow SmoothNewFilledRegions\left(M_{R}\right)$// Algorithm 36:
$M_{R}\leftarrow CutInvalidRegions\left(M_{R},30^{\circ }\right)$
7:
$M_{R}\leftarrow FillHoles\left(M_{R}\right)$
8:$M_{R}\leftarrow SmoothNewFilledRegions\left(M_{R}\right)$ // Algorithm 39:
$M_{f}\leftarrow SurfaceRepair\left(M_{R}\right)$
10:
$Return\;M_{f}$
11:
End



**Figure 2:**
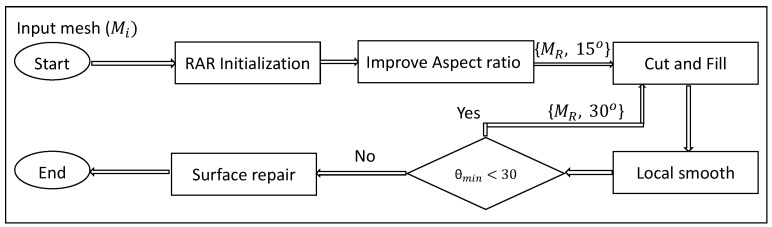
Pipeline of the proposed molecular remeshing method.

Figure 2 shows the pipeline of the proposed method. In the first step, the input model is remeshed with RAR. In the second step, the aspect ratios of the mesh are improved using Algorithm 2. In the third step, the local regions around the minimal angle with threshold $\theta_{min}$ = 15${}^{\circ }$ are removed. In the next step, the holes created are refilled. In the fifth step, the newly filled regions are smoothed locally (Algorithm 3). Similarly, these last three steps (i.e., cut, fill, and smooth) are again repeated with threshold $\theta_{min}$ = 30${}^{\circ }$. Finally, the mesh is passed through a surface repair module to avoid any intersecting faces or non-manifoldness (if any). The algorithm is further described in the following subsections.

### 4.1. Initialization with RAR Method

The input mesh has many zero degree angles and redundant vertices. Initially, we apply the RAR method to improve the input mesh. We select RAR [10] for remeshing because this method is comparatively easy to control, simple to implement, and computationally efficient. RAR uses Equation (8) as an adaptive sizing function to compute the edge length $L(e_{i})$ for an edge $e_{i}$ with vertices v1 and v2 at its two ends.

(8) $$L\left(e_{i}\right)=min\left\{L\left(v_{1}\right),L\left(v_{2}\right)\right\}$$

where the sizing field $L(v_{i})$ is calculated for vertex $v_{i}$ using Equation (9):

(9) $$L\left(v_{i}\right)=\sqrt{6\varepsilon /\kappa_{i}-3\varepsilon^{2}}$$

where ε is error tolerance, and $\kappa_{i}$ is the maximum absolute curvature of a vertex. The maximum absolute curvature $\kappa_{i}$ for a vertex $v_{i}$ is calculated from the mean curvature $H_{i}$ and Gaussian curvature $K_{i}$ using Equations (10)–(12) [52]:

(10) $$\kappa_{i}=H_{i}+\sqrt{H_{i}^{2}-K_{i}}$$

(11) $$K_{i}=\frac{1}{A_{i}}+\left(\pi -\sum_{j\in N(i)}\theta_{i}\right)$$

(12) $$H_{i}=\frac{1}{2}∥\Delta v_{i}∥$$

Here, $\theta_{i}$ represents the adjacent triangle angles around a vertex $v_{i}$, and $A_{i}$ represents the corresponding Voronoi area.

An edge with shorter actual length than $\frac{4}{5}L$ collapses and splits if its actual length is longer than $\frac{4}{3}L$. The RAR method improves the quality for most of the triangles, but it fails to remove all zero degree angles from a raw input molecular mesh and we need further improvements.

### 4.2. Aspect Ratio Improvement

After pre-processing with RAR we use a global smoothing method to improve the aspect ratios of the triangles. Algorithm 2 improves the aspect ratios and is described below.

**Algorithm 2** Improve Aspect Ratio ($M_{R}$)
1:**for** each vertex $v_{i}\in M_{R}$ **do**2:     $v_{f}\leftarrow CalculateNewPositionForVertex(v_{i})$3:     **if** $AR_{v_{i}}>AR_{v_{f}}$ **then**4:        $v_{i}\leftarrow v_{f}$ // Translate the vertex5:     **end if**6:
**end for**
7:
$Return\;M_{R}$
8:
End



For each vertex, we calculate the new position as the center of mass of the corresponding Voronoi cell in the same manner as CVT [53] and then as the Laplacian center (Equation (1)) of the one-ring neighbourhood. The Laplacian method [32] is used for computing the new position $v_{f}$ as the median position of its one-ring neighbors using Equation (1).

Equation (13) computes aspect ratio for vertex *v* as the average of the aspect ratios of the adjacent triangles. Equation (14) computes aspect ratio for a single triangle *t*. The aspect ratio is computed for vertex new position as well as its original position using Equation (13). The vertex is translated to the new position if it improves the aspect ratio, otherwise translation is skipped. This process of aspect ratio improvement is repeated on all vertices of the mesh.

(13) $$AR_{v}=\frac{1}{N}\sum_{t\in T}AR\left(t\right)$$

(14) $$AR\left(t\right)=\frac{abc}{8(S-a)(S-b)(S-c)}$$

where *T* represents the triangles set in the one-ring neighborhood of vertex *v*, and *N* represents the number of triangles in the one-ring neighborhood of vertex *v*, whereas *a*, *b*, and *c* are the lengths of the triangle’s edges and $S=\frac{a+b+c}{2}$.

### 4.3. The Cut-and-Fill Strategy

**Figure 3:**
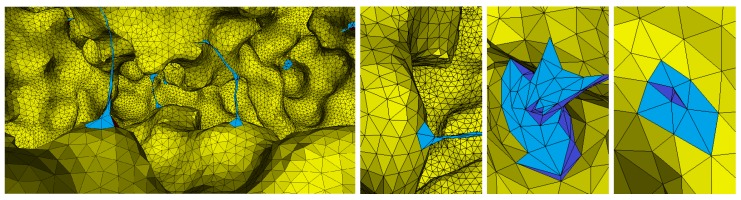
Inside view of a molecular mesh. Blue indicates regions to be cut and filled.

The input mesh has a number of redundant vertices and very tiny angle triangles. The mesh smoothing methods and the edge based operations (edge splitting, edge collapsing, and edge flipping) fail to handle these tiny angles. Therefore, we use a cut & fill strategy to handle this issue. Figure 3 shows the invalid regions in a mesh to be cut and refilled.

**Figure 4:**
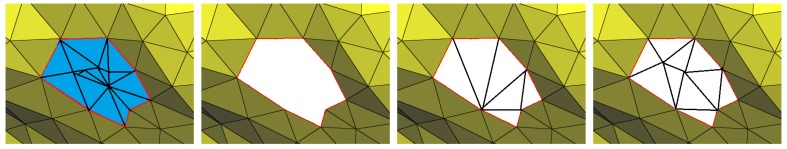
The cut-and-fill strategy to handle invalid regions. From left to right: Invalid regions with low-quality triangles are highlighted in blue, a cut operation is applied, the hole created is filled by directly connecting the boundary vertices, and the newly filled region is locally processed for further quality improvements.

**Figure 5:**
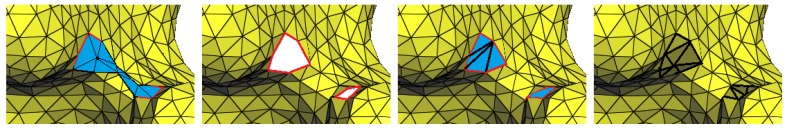
The cut-and-fill strategy for removing tiny regions. From left to right: Narrow region of small triangles (blue color), the cut operation makes holes in the mesh, holes are filled, and the filled regions are smoothed.

Each triangle with an angle $<\theta_{min}$ is labeled as a small triangle. The one-ring neighborhood around each vertex of a small triangle is included in the local regions for the cut-and-fill operations. Each small triangle with its local region around is removed and the holes are refilled (see Figure 4 and Figure 5). Small holes are filled by connecting the boundary edges of the hole, whereas for filling large holes new vertices are also inserted inside the hole. The boundary vertices of each hole are stored in a queue. For the four adjacent vertices $V_{0}$, $V_{1}$, $V_{2}$, and $V_{3}$, we select from center vertex $v_{1}$. The side vertices $V_{1}$ and $V_{3}$ are connected with each other if the minimal angle for the new triangle is less than 15${}^{\circ }$; otherwise, the connection is skipped. The same step is repeated for connecting $V_{1}$ and $V_{0}$. If the connection is not possible, the vertex is changed, and the process is repeated again. In other words, each *i*th vertex of the boundary vertices is connected with the (i+2)th vertex if it does not create angle smaller than 15${}^{\circ }$. If an edge is larger than $0.7(\bar{e})$, where $\bar{e}$ represents the average edge length calculated from the triangles adjacent to the hole. Edge splitting in the later local smoothing also creates new vertices inside the holes. Small tiny regions are created during the mesh processing which are removed with the cut-and-fill method. Figure 5 shows a narrow region of tiny triangles connected at two sides of the surface mesh (blue color). The narrow region is removed making two holes on both sides. The holes are filled and smoothed independently.

### 4.4. Local Smooth

The cut-and-fill strategy eliminates tiny triangles and redundant vertices. However, the newly filled regions still need smoothing for further improvements of the minimal angle. Unlike RAR, our method has a local smoothing module to improve the minimal angles locally. Our method can eliminate all small angles, as shown in Section 5, whereas the RAR method does not eliminate all angles smaller than 30${}^{\circ }$. Algorithm 3 smooths the newly filled regions locally. In the following, we describe the local smoothing method of the algorithm.

**Algorithm 3** Smooth New Filled Regions ($M_{R}$)
1:Collapse($M_{R}$)2:Split($M_{R}$)3:**for** each vertex $v_{i}\in NewFilledRegions$ **do**4:     $c_{i}\leftarrow \frac{1}{n}\sum_{j=0}^{n-1}q_{j}$5:     **for** $\delta d=1,\frac{1}{2},\frac{1}{4},\frac{1}{6},\ldots ,\epsilon$ **do**6:         $v_{f}\leftarrow c_{i}+k\cdot \delta d$7:         **if** (Vertex translation improves minimal angle) **then**8:             $v_{i}\leftarrow v_{f}$ // Translate the vertex9:         **end if**10:     **end for**11:
**end for**
12:
$Return\;M_{R}$
13:
End



In the first step, we collapse the short edges (an edge with opposite angle <30${}^{\circ }$) to remove small angles. In the second step, we split the long edges (an edge with opposite angle >90${}^{\circ }$). The remaining steps are applied to each vertex in the newly filled regions. In the fourth step, the Laplacian center of the one ring around the vertex is calculated. Each smoothing iteration calculates the new position $v_{f}=c_{i}+k\cdot \delta d$ near the Laplacian center $c_{i}$. Here, δd represents step size, which is a small distance (to be added with $c_{i}$), whereas *k* is the direction of the vertex translation. The direction *k* is randomly selected to calculate the new position at a distance δd from the vertex’s current position, which could be toward the left, right, up, or down side with respect to the current position. In each iteration, the vertex is translated to the new position $v_{f}$, if it improves the minimal angle.

### 4.5. Surface Repair

We have used constraints for applying meshing operators, such as minimal angle improvements and aspect ratio improvements, which prohibit meshes from creating new defects. However, due to complex structures of molecular meshes, some defects may still exist, causing failure in volume mesh generation by TetGen. Here, we used the “surface repair” module from Graphite [44] to avoid self-intersection and other possible defects.

## 5. Experimental Study

In this section, we present the experimental results of the study. We performed the experiments using an Intel Core i7 3.60 GHz with 16 GB RAM on a 64-bit Windows 7 operating system.

### 5.1. Mesh Quality Analysis

We measured quality in terms of $Q_{min}$ and $Q_{avg.}$, which represents the minimal and average quality of triangle(s), respectively. The quality is calculated for each triangle *t* as $Q\left(t\right)=\frac{6}{\sqrt{3}}\frac{A_{t}}{p_{t}h_{t}}$, where $A_{t}$ is the area of the triangle *t*, $p_{t}$ is its half-perimeter, and $h_{t}$ is the length of its longest edge [54]. Similarly, minimal and maximal angles repressed by $\theta_{min}$ and $\theta_{max}$, respectively, are also used in comparison. In addition, ${\bar{\theta }}_{min}$ representing the average value of the minimum angles in each triangle, the percentage ratio of the triangles with (angle < 30${}^{\circ }$), and the percentage ratio of the regular vertices are also measured for result evaluations. A regular vertex has a valance of 6 for interior vertices and 4 for boundary vertices. In our experiments, we count a vertex as a regular vertex if it has a valance of 5, 6, or 7 for interior vertices and a valance of 3, 4, or 5 for boundary vertices. Furthermore, the aspect ratios (min, max) are computed using Equation (14). We also measured genus using Equation (15) [55]:

(15) $$Genus\left(S\right)=1-\frac{1}{2}E\left(S\right).$$

Here, E(S) represents the Eural number of surface *S*, computed as in Equation (16).

(16) E(S)=nv+nf−ne

where nv, nf, and ne represent the number of vertices, the number of faces, and the number of edges, respectively. Some surfaces may have a negative genus (one cavity causes −1 in genus). For example, for nf=4824, ne=14,460, and nf=9640 (a surface mesh of 1MAG); the genus is −1.

### 5.2. Experiments and Results

**Figure 6:**
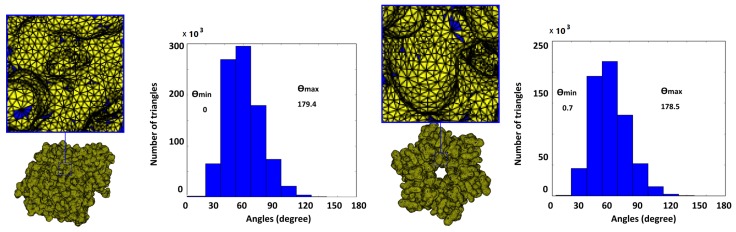
Results of the RAR method. Blue indicates triangles with angles <30${}^{\circ }$. Top: AChE (with $\theta_{min}$ = 0${}^{\circ }$, and 386 pairs of self-intersecting triangles); bottom: Connexin (with $\theta_{min}$ = 0.7${}^{\circ }$, and 36 pairs of self-intersecting triangles).

First, we remeshed two molecular surface meshes using the RAR method. The purpose of this simple experiment was to examine the applicability of the RAR method in molecular remeshing. Figure 6 shows the results of the RAR method, which still have very small angles. To the best of our knowledge, the RAR method has no history in molecular remeshing. In addition, the molecular remeshing results of the RAR method has very small triangles, self-intersections, and fails with AFMPB and TetGen, so we did not select it for detailed comparison. Instead, we selected four existing methods: ISO2Mesh, the Taubin method [33], Graphite [44], and SMOPT for detailed comparison. We selected Laplacian smoothing in the smoothsurf module of ISO2mesh and iterated it 100 times for each experiment.

The Taubin method [33] is implemented in Meshlab [40]. Meshlab provides a number of surface repair modules and smoothing methods [41,42,43]; among which we select the Taubin method [33], which gives comparatively good results. In Graphite, we used the same number of points as the input mesh; all the remaining parameters in default values including CVT smoothing with five Lloyd [53] iterations and 37 Newton [35,53] iterations. In addition to CVT smoothing, we also used the surface repair module of Graphite to remove holes, self-intersections, and other possible defects.

**Figure 7:**
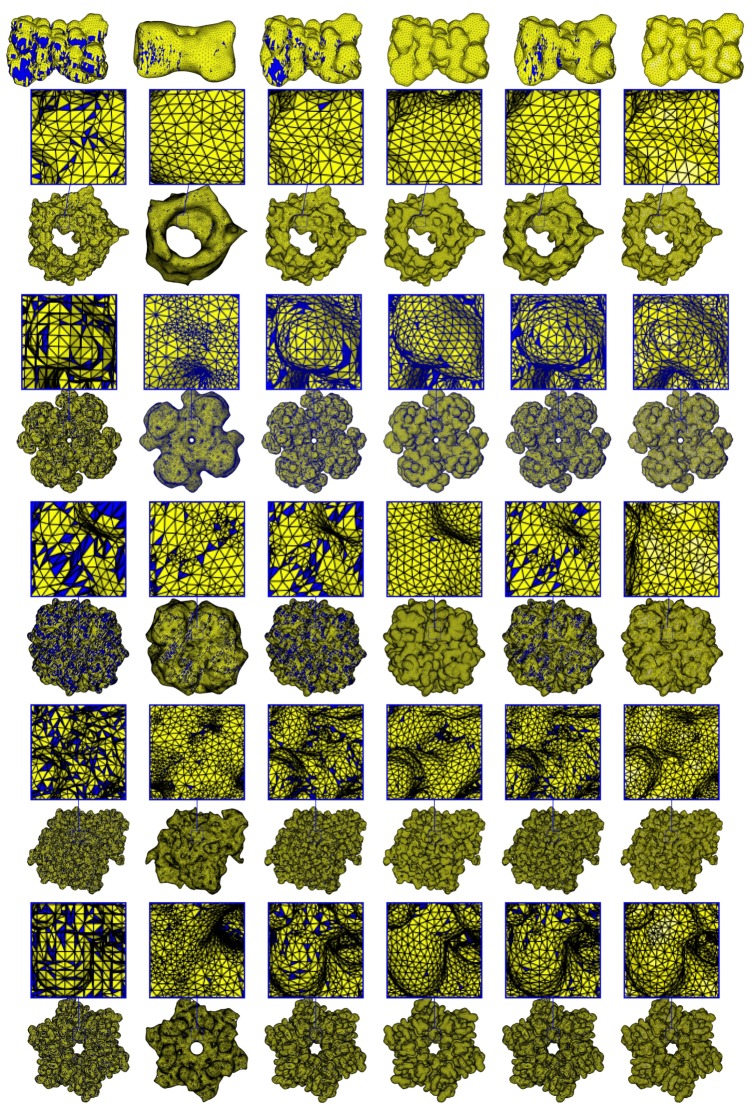
Remeshing results. Blue indicates triangles with angle(s) smaller than 30${}^{\circ }$. From left to right: the input mesh, ISO2mesh, the Taubin method, Graphite, the SMOPT method, and our method. From top to bottom, PDB IDs/molecular names: 1MAG, 2JK4, 1bl8, NaR1R4, AChE, and Connexin.

**Figure 8:**
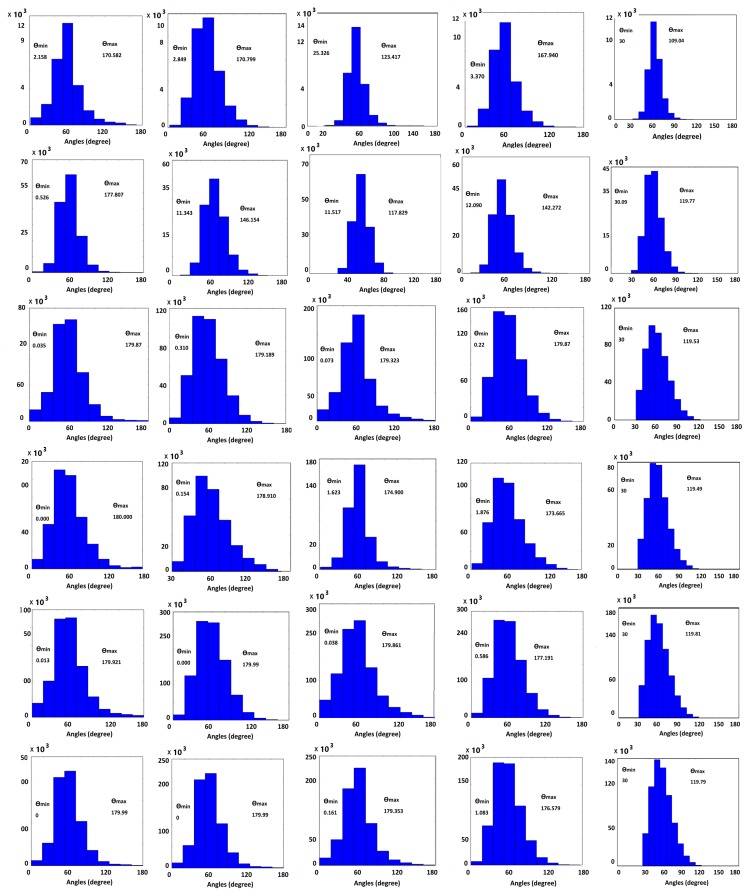
Angle histograms for remeshing results. In each histogram, the *x*-axis represents angles in degrees and the *y*-axis represents the number of triangles. From left to right: ISO2mesh, the Taubin method, Graphite, the SMOPT method, and our method. From top to bottom, PDB IDs/molecular names: 1MAG, 2JK4, 1bl8, NaR1R4, AChE, and Connexin. The histograms for the input meshes are shown in Figure 9.

Figure 7 shows the meshes generated by our method and those generated by ISO2Mesh, the Taubin method, Graphite, and SMOPT, with corresponding. Similarly, Figure 8 shows the corresponding angle histograms of the meshing results. Further quantitative analysis of the result is given in the following subsections.

**Figure 10:**
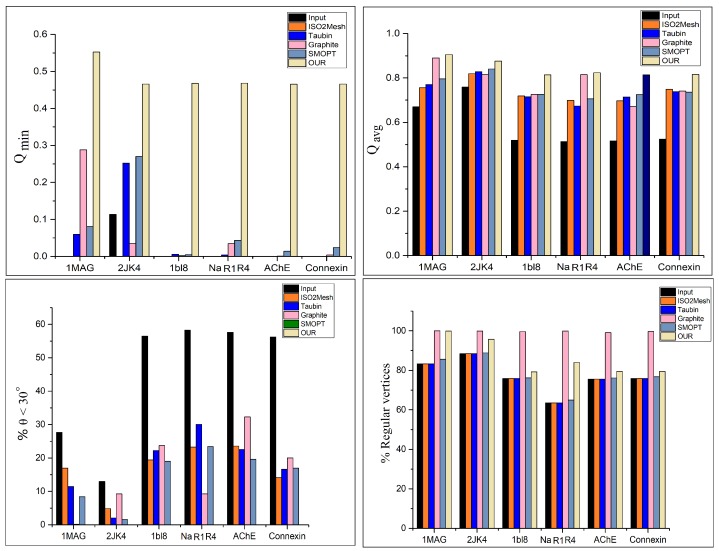
Analysis of meshing quality. Molecular surface meshes with respect to all methods are given in Table 1. Top: $Q_{min}$ and $Q_{avg}$. In both cases, our results have higher values. Bottom left: Triangles with angles less than 30${}^{\circ }$ (%). Our method has no angle smaller than 30${}^{\circ }$. Bottom right: Regular vertices (%). Graphite and our method have higher percentages of regular vertices.

Table 1 contains the statistical results of the experiments, which shows that our method shows a significant improvement in meshing quality over the existing methods. Figure 10 shows the charts of $Q_{min}$, $Q_{avg}$, and % values of the $\theta <30^{\circ }$ and % values of regular vertices. Our method shows a significant improvement over the state-of-the-art methods. Though Graphite performs better than our method in terms of the % values of the regular vertices, our method still shows significant improvement over the other previous methods.

#### 5.2.1. Numerical Simulation Using the Boundary Element Method

**Figure 11:**
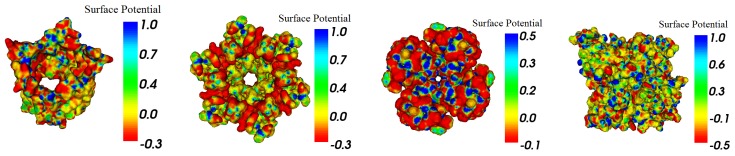
Electrostatic potential on molecular surfaces, calculated with AFMPB. From left to right: 2JK4, connexin, 1bl8, and AChE.

We tested the meshes in the usage of a boundary element method to calculate the Poisson–Boltzmann electrostatics. The BEM software used is a publicly available PB solver AFMPB [56]. MSMS [51] meshes have already been used in many previous boundary element PB works for smaller molecules and have been demonstrated to generate reasonable results. The AFMPB results from meshes of TMSmesh and MSMS were compared. Our test cases (Figure 11) show that AFMPB can undergo and produce converged results. Figure 11, created using VCMM (Visual Continuum Molecular Modeling) tool [57], shows the computed electro-static potentials mapped on the molecular surface.

#### 5.2.2. Shape Preservation and Further Results Analysis

In order to ensure the applicability of our method and shape preservation, we compared our method in terms of area, volume, genus, the number of self-intersecting triangles, TetGen operation, and AFMPB (solvation energy). The numerical results of these terms are given in Table 2, which shows that our approach performs intermediately in comparison to the previous methods in volume and area preservations. In comparison to our method, the Taubin method and Graphite achieve superior area/volume preservations, while ISO2mesh and SMOPT achieve lower area/volume preservations. Hence, our results are satisfactory in area/volume preservations. In addition, our method has no self-intersections of triangles and always outperforms with TetGen and AFMPB. As for solvation energy calculations, a primary requirement for all meshing tools when using AFMPB is to achieve convergent results. As SMOPT, other than any other software studied in this work, was specifically designed for such a goal, and has been validated in terms of both geometry feature preservations and solvation energy calculations in previous work [6,9], the results of the energy calculations using SMOPT in this work can be used as a reference with respect to other meshing tools. Considering this, the solvation energy calculation results using our new mesh refinement approach are overall closer to the SMOPT results relative to the Taubin and Graphite results.

**Figure 12:**
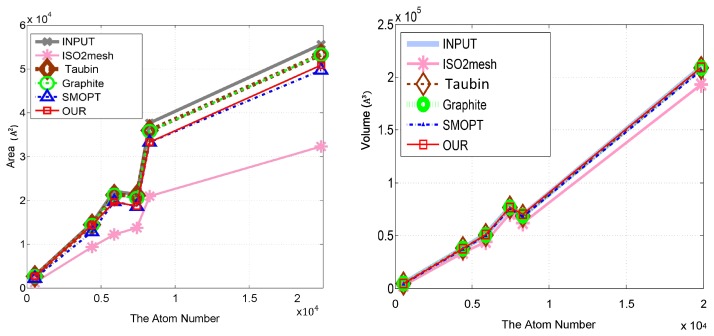
Area and volume of the initial meshes and of the improved meshes generated by our method and the other methods for the molecules in Table 2. Left: area; right: volume.

**Figure 13:**
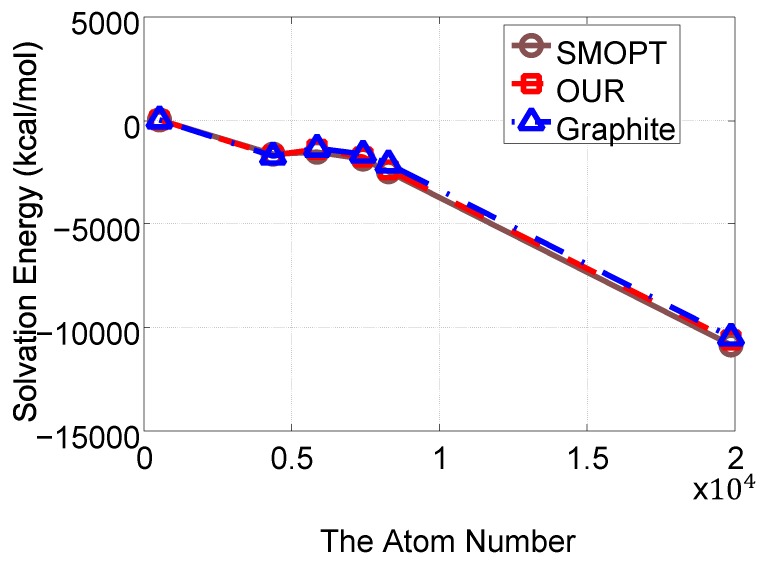
Solvation energy between the improved meshes generated by SMOPT, Graphite, and our method for the molecules in Table 2.

**Figure 14:**
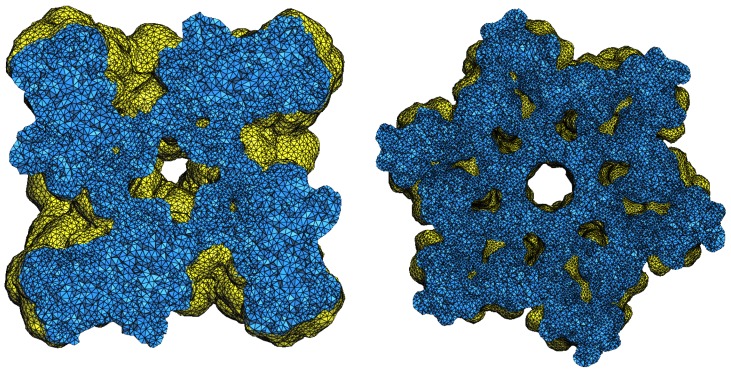
Cut views of tetrahedral meshes. Left: 1bl8; right: Connexin.

Figure 12 plots the volumes and areas of the meshes of the input and improved meshes. The plots show that our method, compared with the other methods, causes a minor deviation in area and volume. Figure 13 plots the solvation energies calculated for our results as well as SMOPT and shows that the results of both methods are similar. The input mesh and the results of ISO2mesh usually fail with solvation energy due to the low-quality elements such as self-intersecting triangle pairs. The results improved by our method can be further used for tetrahedral mesh generation. An example of tetrahedral meshes is shown in Figure 14.

## 6. Conclusions

We have here presented a simple yet robust method for quality molecular surface meshing. Our method starts with RAR initialization and is followed by aspect ratio improvement and a cut-and-fill strategy with local operators to handle existing defects. Our method achieves a significant improvement in minimal and maximal angles and other meshing quality parameters. In addition, our method is able to eliminate existing defects such as self-intersections, redundant vertices, and holes. In terms of regular vertices and the preservation of area and volume, some of the previous methods have better results than our method, but the results of our method are still satisfactory. We plan to improve the efficiency of our method and to produce a software product for end-users. We also plan to introduce a mesh generation method for extracting surface meshes (with minimal defects) from PQR files in an efficient and robust manner, and to contribute to tetrahedral generation for molecular surface meshes. 

## Figures and Tables

**Figure 9:**
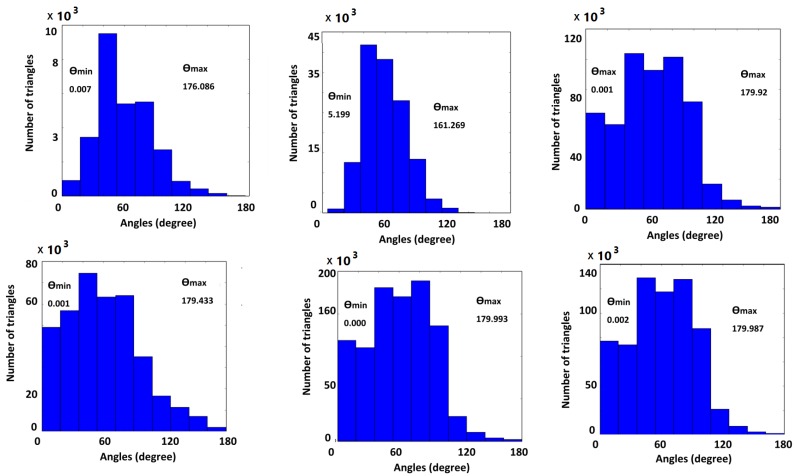
Angle histograms for the input meshes. From top-left to bottom-right, PDB IDs/molecular names: 1MAG, 2JK4, 1bl8, NaR1R4, AChE, and Connexin.

**Table 1:** Comparative surface remeshing results. Our method shows a significant improvement in mesh quality. Note: In Graphite, we used the CVT method [35,53]. The angles are measured in degrees. The model names are the PDB IDs/molecular names. The term undef. represents the undefined value.

Model	Method	#v	$Q_{\min}$	$Q_{\mathrm{avg}}$	$\theta_{\min}$	${\bar{\theta }}_{\min}$	$\theta_{\max}$	$\theta <30^{\circ }$	Reg. v’s	${\mathrm{AR}}_{\max}$	${\mathrm{AR}}_{\mathrm{avg}}$
1MAG	Input	4824	0.0002	0.6701	0.0069	36.41	176.09	27.67%	83.31 %	undef.	undef.
	ISO2MESH	4824	0.0	0.7558	0	41.7	170.58	16.97 %	83.31%	undef.	undef.
	Taubin	4824	0.0595	0.7700	2.85	42.53	170.80	11.47%	83.31%	undef.	undef.
	Graphite	4811	0.4316	0.9024	25.32	52.15	123.42	0.10%	99.58%	2.41	1.04
	SMOPT	4718	0.0808	0.7958	3.37	44.19	167.94	8.42 %	85.59%	51.30	1.21
	OUR	4719	0.5523	0.9054	30.52	52.30	109.043	0%	99.84%	1.67	1.03
2JK4	Input	23,312	0.1137	0.75867	5.20	41.51	161.27	12.97%	88.41%	23.71	1.23
	ISO2MESH	23,312	0.0	0.8196	0.53	45.70	177.81	4.85%	88.41%	undef.	undef.
	Taubin	23,312	0.2523	0.8280	11.34	46.41	146.15	2.04%	88.41%	6.16	1.12
	Graphite	23,106	0.2880	0.8899	11.53	50.84	117.83	0.16%	99.97%	3.40	1.05
	SMOPT	23,139	0.2700	0.8402	12.09	47.22	142.27	1.53 %	88.86%	5.24	1.10
	OUR	23,134	0.4660	0.8762	30.09	49.97	119.77	0%	95.73%	2.14	1.05
1bl8	Input	85,904	0.0	0.5191	0.0	26.30	179.92	56.46%	75.84%	undef.	undef.
	ISO2MESH	85,904	0.0	0.7190	0.03	39.31	179.87	19.39 %	75.84%	undef.	undef.
	Taubin	85,904	0.0058	0.7147	0.31	38.24	179.19	22.22%	75.84%	undef.	undef.
	Graphite	85,466	0.0020	0.7262	0.07	39.66	179.32	23.71%	99.57%	undef.	undef.
	SMOPT	85,601	0.0046	0.726	0.22	39.20	179.87	19.04%	76.19%	undef.	undef.
	OUR	74,623	0.4679	0.8141	30.0	45.31	119.53	0.0%	79.27%	2.13	1.12
NaR1R4	Input	63,310	0.0	0.5135	0.0	26.319	179.43	58.21%	63.51%	undef.	undef.
	ISO2MESH	63,310	0.0	0.6991	0.0	37.92	180	23.26 %	63.51%	undef.	undef.
	Taubin	63,310	0.0039	0.6734	0.15	36.30	178.91	30.08 %	63.51%	undef.	undef.
	Graphite	61,247	0.03452	0.8151	1.62	45.56	174.9	9.24%	99.92%	undef.	undef.
	SMOPT	61,129	0.0437	0.7063	1.88	38.35	173.66	23.41 %	64.96%	179.55	1.47
	OUR	57,024	0.4682	0.8233	30.0	45.90	119.49	0%	83.95%	2.12	1.11
AChE	Input	158,088	0.0	0.5162	0.0	25.95	179.99	57.59%	75.58%	undef.	undef.
	ISO2MESH	158,088	0.0	0.6966	0.0	37.88	179.92	23.56 %	75.58%	undef.	undef.
	Taubin	158,088	0.0	0.7141	0.0	38.16	179.32	22.56%	75.58%	undef.	undef.
	Graphite	150,606	0.0008	0.6708	0.04	35.97	179.86	32.27%	99.07%	undef.	undef.
	SMOPT	155,565	0.0140	0.7250	0.59	39.10	177.19	19.62%	76.16%	undef.	undef.
	OUR	124,326	0.4656	0.8142	30.0	45.32	119.81	0.0%	79.45 %	2.14	1.12
Connexin	Input	107,500	0.0	0.5248	0.0	26.53	179.99	56.21%	75.87%	undef.	undef.
	ISO2MESH	107,500	0.0	0.7494	0.0	41.16	179.99	14.10 %	75.87%	undef.	undef.
	Taubin	107,500	0.0	0.7381	0.0	39.84	179.19	16.66%	75.87%	undef.	undef.
	Graphite	104,255	0.0040	0.74075	0.16	40.41	179.35	20.0%	99.64%	undef.	undef.
	SMOPT	105,645	0.0236	0.7364	1.08	39.85	176.58	16.98%	76.78%	614.1	1.46
	OUR	101,574	0.4658	0.8158	30.0	45.44	119.79	0%	79.46%	2.14	1.12

**Table 2:** Results of applying the initial meshes and the improved meshes to AFMPB and TetGen. Note: The unit for area is A${}^{2}$, the unit of volume is A${}^{3}$, and the unit of solvation energy is kcal/mol. The model names are the PDB IDs/molecular names. Here “*√*” means success and “×” means failure of volume mesh generation by TetGen.

Model	Natoms	Method	Area	Volume	Genus	Self-Intersection	TetGen	AFMPB^(solv. energy)^
1MAG	552	Input	2807.82	4531.35	−1	0	*√*	$0.00408\times 10^{3}$
		ISO2mesh	1247.86	2780.61	−1	0	*√*	Failed
		Taubin	2660.44	4515.91	−1	0	*√*	$0.00320\times 10^{3}$
		Graphite	2625.30	4499.45	3	0	*√*	$0.00322\times 10^{3}$
		SMOPT	2195.70	4174.41	3	0	*√*	$0.00263\times 10^{3}$
		Our	2624.00	4499.73	2	0	*√*	$0.00322\times 10^{3}$
2JK4	4393	Input	14,787.54	37,962.87	4	0	*√*	$-1.59041\times 10^{3}$
		ISO2mesh	12,831.09	33,356.03	4	203	×	Failed
		Taubin	14,413.94	38,039.34	4	0	*√*	$-1.69390\times 10^{3}$
		Graphite	14,307.57	37,908.52	5	0	*√*	$-1.68641\times 10^{3}$
		SMOPT	12,770.33	37,284.66	6	0	*√*	$-1.63068\times 10^{3}$
		Our	14,286.88	37,911.41	5	0	*√*	$-1.65811\times 10^{3}$
1bl8	5892	Input	22,094.98	50,919.54	103	0	*√*	Failed
		ISO2mesh	12,191.41	44,064.03	103	263	×	Failed
		Taubin	21,231.87	50,738.14	103	98	*√*	$-1.34769\times 10^{3}$
		Graphite	21,166.78	50,688.84	97	0	*√*	$-1.34629\times 10^{3}$
		SMOPT	19,673.38	49,935.09	103	0	*√*	$-1.50455\times 10^{3}$
		Our	19,629.99	50,901.04	58	0	*√*	$-1.39705\times 10^{3}$
NaR1R4	7443	Input	21,301.15	76,894.66	−1	0	*√*	$-1.54776\times 10^{3}$
		ISO2mesh	13,669.33	70,900.77	−1	17	×	Failed
		Taubin	21,077.51	76,825.02	−1	22	×	$-1.59197\times 10^{3}$
		Graphite	20,480.95	76,807.18	14	0	*√*	$-1.59706\times 10^{3}$
		SMOPT	18,574.04	75,558.13	14	0	*√*	$-1.85589\times 10^{3}$
		Our	18,723.79	76,881.15	8	0	*√*	$-1.74595\times 10^{3}$
AChE	8280	Input	37,653.08	69,058.97	225	0	*√*	Failed
		ISO2mesh	20,956.70	61,715.23	225	433	×	Failed
		Taubin	35,940.34	68,764.13	225	489	×	Failed
		Graphite	35,760.42	68,699.76	341	0	*√*	$-2.07574\times 10^{3}$
		SMOPT	33,222.25	67,940.30	345	0	*√*	$-2.48270\times 10^{3}$
		Our	30,099.40	70,371.76	93	0	*√*	$-2.37904\times 10^{3}$
Connexin	19,883	Input	55,604.89	209,415.59	31	0	*√*	Failed
		ISO2mesh	32,292.15	193,006.51	31	246	×	Failed
		Taubin	53,231.12	208,781.05	31	5	×	Failed
		Graphite	53,272.10	208,731.02	94	0	*√*	$-10.4865\times 10^{3}$
		SMOPT	49,695.69	206,505.02	96	0	*√*	$-10.8436\times 10^{3}$
		Our	50,806.90	209,318.82	68	0	*√*	$-10.6104\times 10^{3}$

## Abbreviations

The following abbreviations are used in this manuscript:

CVT	centroidal Voronoi tessellation
RAR	real-time adaptive remeshing
PDB	protein data bank
AR	aspect ratio
PB	Poisson–Boltzmann
AFMPB	adaptive fast multipole Poisson–Boltzmann

## References

[1] Parulek J., Viola I. Implicit Representation of Molecular Surfaces. Proceedings of the IEEE Pacific Visualization Symposium (PacificVis 2012).

[2] Dias S., Bora K., Gomes A. CUDA-based Triangulations of Convolution Molecular Surfaces. Proceedings of the 19th ACM International Symposium on High Performance Distributed Computing (HPDC ’10).

[3] Chen M., Tu B., Lu B., Zhang Y.J. (2013). Surface Triangular Mesh and Volume Tetrahedral Mesh Generations for Biomolecular Modeling. Image-Based Geometric Modeling and Mesh Generation.

[4] Chen M., Lu B. (2011). TMSmesh: A Robust Method for Molecular Surface Mesh Generation Using a Trace Technique. J. Chem. Theory Comput..

[5] Chen M., Tu B., Lu B. (2012). Triangulated manifold meshing method preserving molecular surface topology. J. Mol. Graph. Model..

[6] Liu T., Chen M., Lu B. (2018). Efficient and Qualified Mesh Generation for Gaussian Molecular Surface Using Adaptive Partition and Piecewise Polynomial Approximation. SIAM J. Sci. Comput..

[7] Attene M., Campen M., Kobbelt L. (2013). Polygon Mesh Repairing: An Application Perspective. ACM Comput. Surv..

[8] Fang Q., Boas D.A. Tetrahedral mesh generation from volumetric binary and grayscale images. Proceedings of the 2009 IEEE International Symposium on Biomedical Imaging: From Nano to Macro.

[9] Liu T., Chen M., Song Y., Li H., Lu B. (2016). Quality improvement of surface triangular mesh using a modified Laplacian smoothing approach avoiding intersection. PLoS ONE.

[10] Dunyach M., Vanderhaeghe D., Barthe L., Botsch M. (2013). Adaptive Remeshing for Real-Time Mesh Deformation. Eurographics Short Papers Proceedings.

[11] Chen M., Lu B. (2013). Advances in biomolecular surface meshing and its applications to mathematical modeling. Chin. Sci. Bull..

[12] Botsch M., Kobbelt L., Pauly M., Alliez P., Levy B. (2010). Polygon Mesh Processing.

[13] Alliez P., Ucelli G., Gotsman C., Attene M. (2008). Recent Advances in Remeshing of Surfaces. Shape Analysis and Structuring.

[14] Bade R., Haase J., Preim B. (2006). Comparison of fundamental mesh smoothing algorithms for medical surface models. Simul. Vis..

[15] Heckbert P., Garland M. (1997). Survey of Polygonal Surface Simplification Algorithms.

[16] Liu Y.J., Xu C., Fan D., He Y. (2015). Efficient construction and simplification of Delaunay meshes. ACM Trans. Graph..

[17] Cheng S.W., Dey T.K., Shewchuk J.R. (2012). Delaunay Mesh Generation.

[18] Schreiner J., Scheidegger C.E., Fleishman S., Silva C.T. (2006). Direct (Re)Meshing for Efficient Surface Processing. Computer Graphics Forum.

[19] Lai Y., Jin M., Xie X., He Y., Palacios J., Zhang E., Hu S., Gu X. (2010). Metric-Driven RoSy Field Design and Remeshing. IEEE Trans. Vis. Comput. Graph..

[20] Jakob W., Tarini M., Panozzo D., Sorkine-Hornung O. (2015). Instant field-aligned meshes. ACM Trans. Graph..

[21] Wang Y., Yan D.M., Tang C., Liu X. Obtuse triangle elimination for isotropic remeshing. Proceedings of the ACM SIGGRAPH 2017 Posters.

[22] Marchandise E., Remacle J.F., Geuzaine C. (2014). Optimal parametrizations for surface remeshing. Eng. Comput..

[23] Zhong Z., Shuai L., Jin M., Guo X. (2014). Anisotropic surface meshing with conformal embedding. Graph. Models.

[24] Valette S., Chassery J.M., Prost R. (2008). Generic remeshing of 3D triangular meshes with metric-dependent discrete Voronoi Diagrams. IEEE Trans. Vis. Comput. Graph..

[25] Boissonnat J.D., Oudot S. (2005). Provably Good Sampling and Meshing of Surfaces. Graph. Models.

[26] Yan D.M., Bao G., Zhang X., Wonka P. (2014). Low-Resolution Remeshing Using the Localized Restricted Voronoi Diagram. IEEE Trans. Vis. Comput. Graph..

[27] Liu Y.J., Xu C.X., Yi R., Fan D., He Y. (2016). Manifold Differential Evolution (MDE): A Global Optimization Method for Geodesic Centroidal Voronoi Tessellations on Meshes. ACM Trans. Graph..

[28] Ahmed A., Guo J., Yan D.M., Franceschi J.Y., Zhang X., Deussen O. (2017). A Simple Push-Pull Algorithm for Blue-Noise Sampling. IEEE Trans. Vis. Comput. Graph..

[29] Alliez P., Meyer M., Desbrun M. (2002). Interactive Geometry Remeshing. ACM Trans. Graph..

[30] Edwards J., Wang W., Bajaj C.L. Surface Segmentation for Improved Remeshing. Proceedings of the 21st International Meshing Roundtable (IMR 2012).

[31] Hu K., Yan D.M., Bommes D., Alliez P., Benes B. (2017). Error-Bounded and Feature Preserving Surface Remeshing with Minimal Angle Improvement. IEEE Trans. Vis. Comput. Graph..

[32] Field D.A. (1988). Laplacian Smoothing and Delaunay Triangulations. Commun. Appl. Numer. Methods.

[33] Taubin G. (1995). A Signal Processing Approach to Fair Surface Design. Proceedings of the 22nd Annual Conference on Computer Graphics and Interactive Techniques (SIGGRAPH ’95).

[34] Du Q., Faber V., Gunzburger M. (1999). Centroidal Voronoi tessellations: Applications and algorithms. SIAM Rev..

[35] Liu Y., Wang W., Lévy B., Sun F., Yan D.M., Lu L., Yang C. (2009). On Centroidal Voronoi Tessellation—Energy Smoothness and Fast Computation. ACM Trans. Graph..

[36] Mansouri S., Ebrahimnezhad H. (2016). Segmentation-based semi-regular remeshing of 3D models using curvature-adapted subdivision surface fitting. J. Vis..

[37] Yan D.M., Wonka P. (2016). Non-Obtuse Remeshing with Centroidal Voronoi Tessellation. IEEE Trans. Vis. Comput. Graph..

[38] Ju T. (2004). Robust Repair of Polygonal Models. ACM Trans. Graph..

[39] Attene M. (2010). A lightweight approach to repairing digitized polygon meshes. Vis. Comput..

[40] Cignoni P., Callieri M., Corsini M., Dellepiane M., Ganovelli F., Ranzuglia G., Scarano V., Chiara R.D., Erra U. (2008). MeshLab: An Open-Source Mesh Processing Tool. Eurographics Italian Chapter Conference.

[41] Yutaka A.B., Ohtake E.B.Y. A Comparison of Mesh Smoothing Methods. Proceedings of the Israel-Korea BiNational Conference on Geometric Modeling and Computer Graphics.

[42] Vollmer J., Mencl R., Müller H. (1999). Improved Laplacian Smoothing of Noisy Surface Meshes. Comput. Graph. Forum.

[43] Pietroni N., Tarini M., Cignoni P. (2010). Almost isometric mesh parameterization through abstract domains. IEEE Trans. Vis. Comput. Graph..

[44] Graphite. http://alice.loria.fr/index.php/software/3-platform/22-graphite.html.

[45] Decherchi S., Rocchia W. (2013). A general and Robust Ray-Casting-Based Algorithm for Triangulating Surfaces at the Nanoscale. PLoS ONE.

[46] Liu T., Chen M., Lu B. (2015). Parameterization for molecular Gaussian surface and a comparison study of surface mesh generation. J. Mol. Model..

[47] Sakalli I., Schöberl J., Knapp E.W. (2014). mFES: A Robust Molecular Finite Element Solver for Electrostatic Energy Computations. J. Chem. Theory Comput..

[48] Dolinsky T.J., Nielsen J.E., McCammon J.A., Baker N.A. (2004). PDB2PQR: An automated pipeline for the setup of Poisson–Boltzmann electrostatics calculations. Nucleic Acids Res..

[49] Si H. (2015). TetGen, a Delaunay-Based Quality Tetrahedral Mesh Generator. ACM Trans. Math. Softw..

[50] Lu B., Cheng X., Huang J., Mccammon J.A. (2010). AFMPB: An Adaptive Fast Multipole Poisson-Boltzmann Solver for Calculating Electrostatics in Biomolecular Systems. Comput. Phys. Commun..

[51] Sanner M.F., Olson A.J., Spehner J.C. (1995). Fast and Robust Computation of Molecular Surfaces. Proceedings of the Eleventh Annual Symposium on Computational Geometry (SCG ’95).

[52] Meyer M., Desbrun M., Schröder P., Barr A.H. (2002). Discrete Differential-Geometry Operators for Triangulated 2-Manifolds.

[53] Lloyd S. (1982). Least squares quantization in PCM. IEEE Trans. Inf. Theory.

[54] Frey P., Borouchaki H. Surface mesh evaluation. Proceedings of the 6th International Meshing Roundtable.

[55] Hirzebruch F.E.P., Kreck M. (2009). On the concept of genus in topology and complex analysis. Not. Am. Math. Soc..

[56] Zhang B., Peng B., Huang J., Pitsianis N.P., Sun X., Lu B. (2015). Parallel AFMPB solver with automatic surface meshing for calculations of molecular solvation free energy. Comput. Phys. Commun..

[57] Bai S., Lu B. (2014). VCMM: A visual tool for continuum molecular modeling. J. Mol. Graph. Model..

